# Peri-lead edema in deep brain stimulation: common complication or rare challenge?

**DOI:** 10.3389/fsurg.2025.1591985

**Published:** 2025-08-21

**Authors:** Martin Nevrly, Filip Blazek, David Krahulik, Pavel Otruba, Eva Cechakova, Ahmed Naser Mohamed Afifi, Petr Kanovsky

**Affiliations:** ^1^ Department of Neurology, University Hospital and Faculty of Medicine and Dentistry, Palacký University, Olomouc, Czechia; ^2^ Department of Neurosurgery, University Hospital and Faculty of Medicine and Dentistry, Palacký University, Olomouc, Czechia; ^3^ Department of Radiology, University Hospital and Faculty of Medicine and Dentistry, Palacký University, Olomouc, Czechia

**Keywords:** peri-lead edema, deep brain stimulation, neurological complications, corticosteroid therapy, postoperative monitoring, pseudocyst

## Abstract

**Introduction:**

Peri-lead edema (PLE) is a commonly observed but often asymptomatic complication of deep brain stimulation (DBS). While usually transient and benign, severe cases of PLE can result in neurological symptoms, impacting patient outcomes. This case series explores the clinical course, management, and outcomes of symptomatic PLE in a series of five patients undergoing DBS.

**Objective:**

To analyze the presentation and management strategies for symptomatic peri-lead edema identified in patients undergoing DBS for movement disorders.

**Methods:**

A retrospective review of 191 patients who underwent DBS at the University Hospital in Olomouc, Czech Republic, between 2008 and 2024 was conducted. Postoperative imaging and clinical follow-ups were used to identify and evaluate cases of symptomatic PLE. Patients who developed symptomatic PLE were treated with corticosteroids and monitored through imaging and neurological assessments.

**Results:**

Among the 191 patients, we identified 5 (2.6%) who developed symptomatic PLE characterized by cognitive decline, motor disturbances, and, in some cases, pseudocyst formation. Symptoms typically presented several weeks to months postoperatively. Management with corticosteroid therapy resulted in clinical improvement and resolution of edema in all cases. Following the resolution of PLE, DBS therapy was successfully re-initiated, achieving favorable therapeutic outcomes.

**Conclusions:**

Symptomatic PLE is a rare but clinically significant complication of DBS. Early detection and timely management with corticosteroids are critical for symptom resolution and successful continuation of DBS therapy. Vigilant postoperative monitoring and further research are essential to improve understanding and management of PLE.

## Introduction

Peri-lead edema (PLE) is a type of localized swelling that occurs around the electrode leads in patients undergoing deep brain stimulation (DBS) therapy (1). This phenomenon is frequently observed on postoperative MRI scans and has been documented in multiple prospective studies (2–4). While PLE is common, it typically remains asymptomatic, presenting only a transient, beneficial lesional effect that may contribute to therapeutic outcomes in the early stages post-surgery. The immediate postoperative microlesional effect, commonly observed intraoperatively or within hours, is distinct from the development of peri-lead edema, which typically manifests days to weeks later and is presumed to be inflammatory in origin rather than a direct mechanical effect.

Despite its usual benign nature, there are cases where PLE manifests more severely, raising clinical concerns (5). Reports in the literature describe instances of significant edema formation leading to larger hypodensities or even cystic changes in the brain tissue surrounding the electrode (6). Such pronounced changes generally appear several months after the DBS implantation, contrasting with the initial, asymptomatic swelling seen immediately postoperatively. Interestingly, these more extensive changes are predominantly unilateral, affecting only one hemisphere of the brain. In these severe cases, patients often experience a noticeable decline in cognitive functions, which can impact quality of life and overall treatment success (7, 8). Fortunately, this cognitive impairment is typically temporary, with most patients showing improvement after a period of observation or following a short course of corticosteroid therapy to reduce inflammation (9).

The occurrence of PLE, particularly in its more extreme manifestations, highlights the importance of vigilant postoperative monitoring in patients who undergo DBS. Close observation allows for the early identification of any abnormal swelling or associated neurological changes, facilitating timely intervention when needed. By managing PLE proactively, healthcare providers can help prevent or mitigate potential adverse effects, supporting a more favorable long-term outcome for individuals receiving DBS therapy (10).

Overall, as DBS therapy becomes increasingly utilized for a variety of neurological conditions (11, 12), understanding and managing complications like PLE is essential to optimizing patient outcomes. Ongoing research is warranted to further elucidate the risk factors, underlying mechanisms, and best practices for managing PLE, especially considering its potential to cause transient but impactful cognitive changes in affected individuals. This understanding will be crucial in advancing DBS treatment protocols and improving patient safety and efficacy in neurostimulation therapies (7). This case series builds on prior reports by presenting a longitudinal clinical and imaging follow-up of symptomatic PLE cases managed using consistent surgical and pharmacologic strategies within a single centre. Our aim is to provide insights into diagnosis, treatment, and recovery patterns, and to suggest practical monitoring and management recommendations for clinicians.

## Materials and methods

Between 2008 and 2024, a total of 191 patients underwent deep brain stimulation (DBS) treatment at the University Hospital in Olomouc, Czech Republic. The primary indications for DBS in this cohort included Parkinson's disease, pharmacoresistant tremor, and dystonia—each representing conditions where conventional treatment options had proven ineffective, warranting the use of neuromodulation for symptom management and quality-of-life improvement. The selection of these patients followed comprehensive clinical assessments to ensure suitability for DBS, including evaluations of disease severity, response to medication, and functional impairments.

The DBS procedure targeted specific brain regions, chosen according to the underlying neurological condition of each patient. For patients with Parkinson's disease, the subthalamic nucleus (STN) (11) was the primary target due to its role in motor control and its established effectiveness in alleviating motor symptoms when stimulated. In cases of pharmacoresistant tremor, the ventrointermedial thalamic nucleus (VIM) was targeted (12), as it is a critical relay center in the tremor circuitry and has shown consistent efficacy in reducing tremor severity. For patients with dystonia, the internal segment of the globus pallidus (GPi) was selected as the target region, given its involvement in motor control pathways and documented success in reducing dystonic movements through DBS.

All surgeries were performed by a single experienced neurosurgeon using a standardized stereotactic technique with microelectrode recording (MER) and intraoperative imaging for targeting accuracy. Implanted electrodes were from the same company in all cases: the first 167 patients, including Cases 1–4, received the Medtronic 3389 model, while the last 24 patients, including Case 5, received directional Medtronic B33005 electrodes. Perioperative sedation protocols and medication were consistent across all cases, and all patients received prophylactic cefazolin. Preoperative and postoperative management protocols were standardized across patients, and no technical deviations or intraoperative complications were reported in these five cases.

Throughout the 16-year period, postoperative imaging, primarily MRI, was routinely conducted to monitor electrode positioning, assess for any complications, and observe any changes in brain tissue, including the presence of peri-lead edema (PLE), which can occasionally develop around the electrode leads. From the total cohort of 191 patients, 5 individuals (approximately 2.6%) developed symptomatic peri-lead edema postoperatively. These patients exhibited neurological symptoms associated with PLE, including cognitive or motor changes that warranted clinical intervention. Symptoms and edema severity were evaluated using imaging studies alongside neurological assessments to determine the clinical impact and appropriate treatment pathway. In all symptomatic cases, contrast-enhanced MRI or CT imaging was performed to exclude alternative causes such as brain abscess or tumor; no pathological contrast enhancement was observed. In our study, “peri-lead edema” refers to T2/FLAIR hyperintense regions surrounding the DBS electrode, without contrast enhancement or signs of infection. “Pseudocyst” denotes a localized, fluid-filled cavity seen on T2 imaging, typically surrounding the lead trajectory, without contrast enhancement or diffusion restriction.

In symptomatic cases, further assessments were conducted to differentiate PLE from other potential complications, with subsequent management tailored to mitigate the edema. Interventions included observation, pharmacological treatment (typically corticosteroids to reduce inflammation), and additional imaging follow-ups to monitor the resolution of edema and symptomatic improvement. The outcomes and recovery trajectories of these 5 cases were documented, contributing to a deeper understanding of PLE's clinical course and the effectiveness of various management strategies in the context of DBS therapy.

From the total cohort of 191 patients, we identified 5 individuals (approximately 2.6%) who developed symptomatic peri-lead edema postoperatively. These cases were identified based on clinical symptoms that prompted imaging; routine systematic imaging of all patients was not performed at fixed intervals postoperatively (Table 1).

**Table 1:** Summary of symptomatic peri-lead edema cases.

Case	Age/Sex	DBS target	Side affected	PLE type	Onset (weeks)	Steroid duration	Outcome
1	59/M	STN (bilateral)	Right	Edema	0.5	2 months	Complete resolution, stimulation resumed
2	59/M	STN (bilateral)	Bilateral	Cystic PLE	12	2 months	Resolution and clinical improvement
3	73/M	STN (bilateral)	Right	Edema	1	2 months	Resolution and stimulation resumed
4	61/M	VIM (Right)	Right	Pseudocyst	6	2 months	Gradual resolution, stimulation delayed
5	59/F	STN (bilateral)	Right	Edema	2	1 month	Resolved; delayed stimulation activation

## Results

This report presents five detailed case studies of patients who developed clinically significant, symptomatic peri-lead edema (PLE) following deep brain stimulation (DBS) surgery. Unlike the commonly observed asymptomatic form of PLE, these cases were characterized by the onset of noticeable neurological symptoms, necessitating medical evaluation and intervention. Each patient presented with a unique profile of cognitive and neurological impairments that could be directly attributed to edema surrounding the implanted DBS electrodes.

The onset of symptoms occurred up to several months postoperatively, distinguishing these cases from the typical, asymptomatic course of PLE, which is generally detected only through imaging and resolves without clinical impact. The symptomatic PLE in these patients manifested as varying degrees of cognitive decline, including issues with memory, attention, and executive function, alongside other neurological deficits such as motor disturbances and emotional lability. These impairments disrupted daily functioning and required careful clinical management to prevent further progression and ensure recovery.

Each case required a tailored approach, combining close observation with pharmacological intervention. Initial assessments included a thorough neurological examination and imaging studies to confirm the presence and extent of edema around the DBS leads. Given the inflammatory nature of the condition, steroid therapy was introduced to reduce edema and alleviate symptoms. The treatment regimen, dosage, and duration of corticosteroid use varied based on each patient's response and symptom severity. In all cases, symptoms gradually regressed with steroid treatment, underscoring the potential of anti-inflammatory therapy to mitigate PLE-related complications effectively. Steroid regimens typically included intravenous dexamethasone (4 mg for each 8 h), followed by an oral taper of dexamethasone (4 mg/day) for next 2 months.

These cases highlight the need for heightened vigilance in monitoring for potential symptomatic PLE, especially in the months following DBS surgery. While asymptomatic PLE is common and typically benign, the occurrence of symptomatic edema can significantly impact patient outcomes if not promptly addressed. These reports emphasize the importance of early detection and intervention in managing symptomatic PLE, advocating for routine imaging and follow-up assessments post-DBS. This approach allows for timely diagnosis and treatment, ultimately enhancing the safety and effectiveness of DBS therapy in patients with complex neurological conditions.

## Case report 1

**Figure 1:**
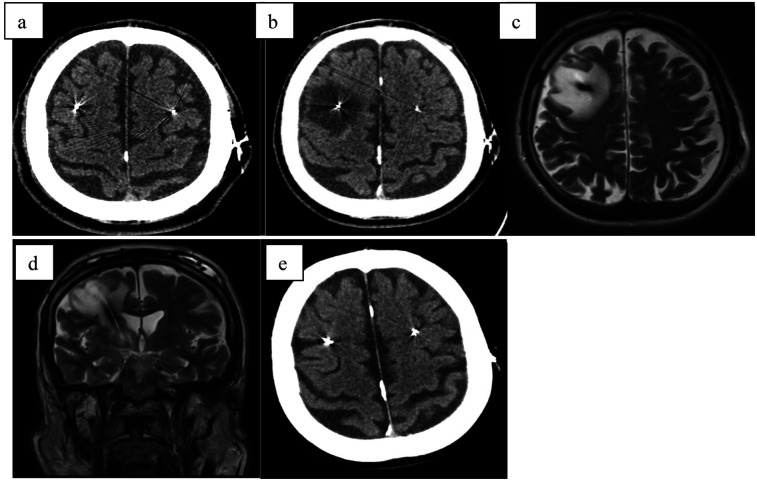
**(a)** CT scan directly after electrode implantation; **(b)** CT scan fourth day after surgery with hypodensity surrounding right electrode; **(c)** MRI-T2 transversal scan 1 week after surgery with large edema surrounding right electrode; **(d)** MRI-T2 coronar scan 1 week after surgery with large edema surrounding right electrode; **(e)** CT scan 3 months after surgery.

A 59-year-old male with a 13-year history of Parkinson's disease underwent bilateral subthalamic deep brain stimulation (DBS) (Figure 1). On the fourth postoperative day, prior to activation of the stimulation, he experienced an acute decline in cognitive function accompanied by torticollis. Imaging studies, including CT and MRI, revealed significant edema surrounding the right electrode. Subsequent examinations ruled out ischemia, hemorrhage, and abscess, and cerebrospinal fluid (CSF) analysis showed no signs of inflammation.

The patient was treated with a course of steroids (dexamethasone 4 mg intravenous every 8 hours for 5 consecutive days with gradual reduction and continuation of 4 mg peroral daily for 2 months), which resulted in substantial improvement in both torticollis and cognitive function within one week. A follow-up CT scan performed six weeks later showed significant regression of the edema. Following this, DBS therapy was initiated, leading to marked improvement in his Parkinson's disease motor symptoms.

## Case report 2

**Figure 2:**
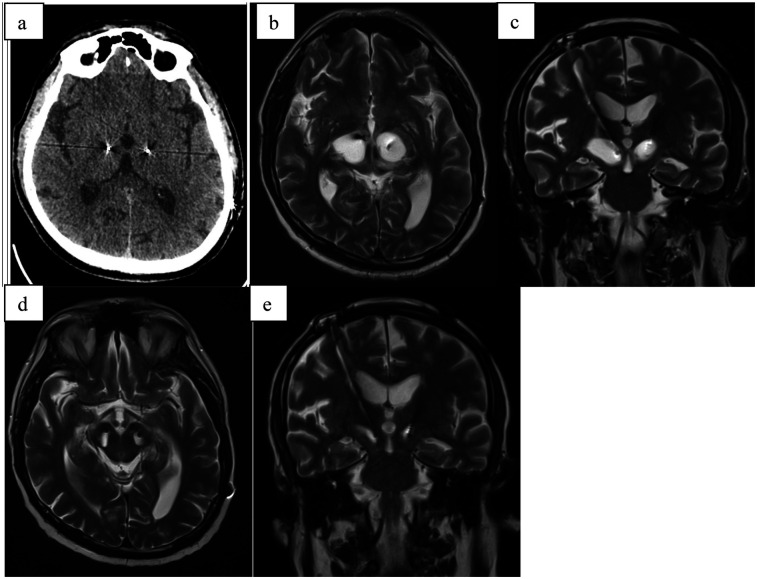
**(a)** CT scan directly after electrode implantation; **(b)** MRI-T2 transversal scan 2 months after stimulation initialization (3 months after surgery) with hyperintensive cystic formation surrounding both electrodes; **(c)** MRI-T2 coronal scan 2 months after stimulation initialization (3 months after surgery) with hyperintensive cystic formation surrounding both electrodes; **(d)** MRI-T2 transversal scan 4 months after stimulation initialization (5 months after surgery) with smaller hyperintensive cystic formation surrounding both electrodes after steroid treatment; **(e)** MRI-T2 coronal scan 4 months after stimulation initialization (5 months after surgery) with smaller hyperintensive cystic formation surrounding both electrodes after steroid treatment.

A 59-year-old male with a six-year history of Parkinson's disease, who had been effectively treated with bilateral subthalamic deep brain stimulation (DBS) for two months, presented with a new onset of symptoms including gait deterioration, restless legs syndrome, urinary incontinence, and dysphonia (Figure 2). Notably, these symptoms persisted regardless of DBS stimulation being turned ON or OFF, indicating they were likely independent of the stimulation settings.

MRI imaging revealed cystic formations surrounding both electrodes, extending into the mesencephalon on the left side. Further investigations ruled out infection and ischemia as potential causes. A course of steroid therapy (same dosage as Case 1) was administered, resulting in partial clinical improvement, with symptomatic relief and a reduction in the size of the cystic formations observed on follow-up imaging. Following the resolution of the cystic formations, stimulation was re-initiated and resulted in sustained clinical improvement, confirming the long-term efficacy of DBS in this case.

## Case report 3

**Figure 3:**
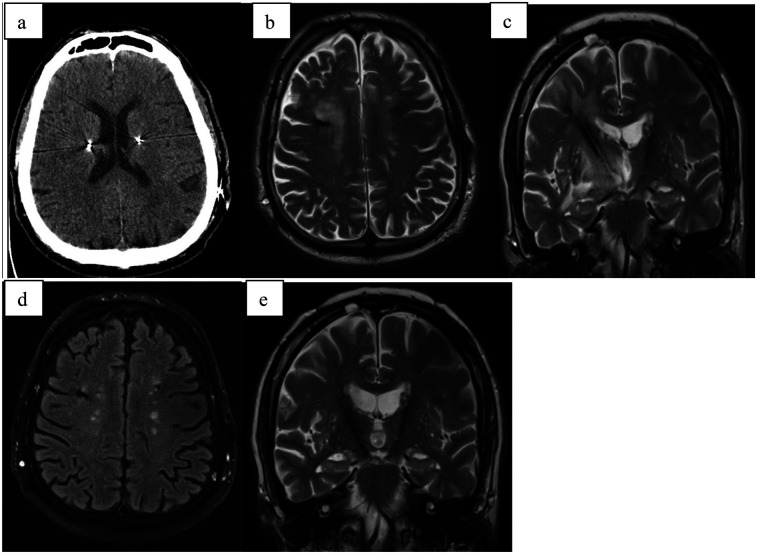
**(a)** CT scan directly after electrode implantation; **(b)** MRI-T2 transversal scan 5 weeks after surgery with hyperintensive cystic formation surrounding right electrode; **(c)** MRI-T2 coronal scan 5 weeks after implant; **(d)** MRI-T2 transversal scan 4 months after surgery with complete regression of edema; **(e)** MRI-T2 coronar scan 4 months after surgery complete regression of edema.

A 73-year-old male with a ten-year history of Parkinson's disease underwent bilateral subthalamic deep brain stimulation (DBS) (Figure 3). One week postoperatively, he developed a gradual decline in mental status, increased rigidity, urinary incontinence, vertigo, and worsening memory. After experiencing these progressively worsening symptoms for three weeks, he sought medical attention and was admitted to the hospital.

Imaging studies, including computed tomography (CT) and magnetic resonance imaging (MRI), revealed significant edema surrounding the right electrode. Following a course of intensive corticosteroid therapy (dosage same as Case 1), a follow-up CT scan demonstrated regression of the edema, and the patient was discharged.

Four weeks later, the patient was readmitted. A control MRI revealed complete resolution of the edema, and DBS therapy was initiated, resulting in marked clinical improvement.

## Case report 4

**Figure 4:**
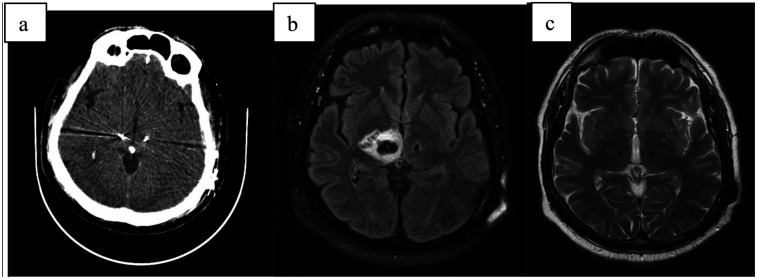
**(a)** CT scan directly after electrode implantation; **(b)** MRI-T2 transversal scan 6 weeks after implant with large pseudocyst around the right electrode; **(c)** MRI-T2 transversal scan 6 months after surgery with complete regression.

A 61-year-old patient with a 40-year history of essential tremor underwent deep brain stimulation (DBS) implantation targeting the ventral intermediate nucleus (VIM) (Figure 4). Following stimulation adjustment, the tremor was well controlled, and the patient expressed satisfaction. However, three weeks after the adjustment, the patient awoke with worsening articulation, tingling sensations, and reduced sensitivity on the left side of the face, as well as decreased dexterity and coordination in the left upper limb. While tremor was absent, the left hand was clumsy, exhibited a leftward pull, and the patient reported unsteadiness while walking.

CT and MRI revealed significant edema and a pseudocyst surrounding the right electrode. Despite intensive corticosteroid and anti-edema therapy (same dosage as Case 1), the pseudocyst continued to increase in size, leading to a progressive decline in clinical status. This included the development of moderate hemiparesis and left-sided hemianopia. Neuroinflammation was ruled out, and the patient was transferred to a rehabilitation unit, which yielded good outcomes.

During this period, the DBS system was activated only on the left electrode, achieving the desired stimulation effect. Follow-up MRI scans and outpatient evaluations showed gradual regression of the pseudocyst, with complete resolution occurring six months post-implantation. Following the disappearance of the pseudocyst, stimulation was successfully activated on the right electrode, achieving full therapeutic effect.

## Case report 5

**Figure 5:**
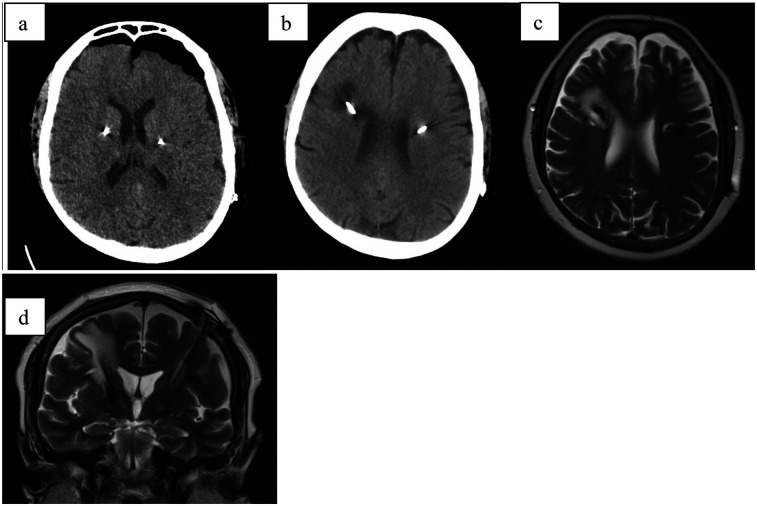
**(a)** CT scan directly after electrode implantation; **(b)** CT scan 4 weeks after implant; **(c)** MRI-T2 axial scan 4 weeks after stimulation initialization showing hyperintensive tissue surrounding the right electrode **(d)** MRI-T2 coronal scan 4 weeks after implant.

A 59-year-old female patient with an 11-year history of Parkinson's disease underwent bilateral deep brain stimulation (DBS) targeting the subthalamic nucleus (STN) (Figure 5). Two weeks post-implantation, the patient was in good clinical condition. However, control CT imaging revealed perifocal edema, and as a result, stimulation was not initiated. The patient was discharged home after starting anti-edematous and corticosteroid therapy (same regimen as Case 1).

During home care, the patient experienced a general deterioration due to internal decompensation of type 2 diabetes mellitus, the onset of a urinary tract infection, and exacerbation of chronic obstructive pulmonary disease (COPD), necessitating hospitalization in the internal medicine department. The dexamethasone was stopped after one month of therapy due to acquired diabetes.

After stabilization of her overall condition, a follow-up MRI was performed three months post-implantation, revealing complete resolution of the edema around the electrode. Stimulation was subsequently activated, achieving excellent therapeutic effect.

## Discussion

The presented retrospective case series provides critical insights into symptomatic peri-lead edema (PLE), a rare but clinically significant complication following deep brain stimulation (DBS). While asymptomatic PLE has been extensively documented and is often transient with minimal clinical impact, these cases illustrate the potential for PLE to progress to a symptomatic and debilitating condition, underscoring the necessity for vigilant postoperative monitoring. The diverse manifestations observed, ranging from cognitive decline and motor dysfunction to the formation of cystic changes, suggest a complex interplay of inflammatory mechanisms localized around the implanted electrodes. PLE was observed in many cases after DBS procedure and in a lot of them is asymptomatic (6). In our cohort was not done control imaging examination in each case, only in symptomatic cases. In all of these symptomatic cases was corticosteroid therapy administered. The observed responsiveness to corticosteroid therapy suggests a potential role of inflammation in the pathophysiology of symptomatic PLE. While previous studies have reported individual cases or heterogeneous series of PLE, our study provides a uniform, longitudinal clinical and imaging assessment from a single institution, highlighting the course and responsiveness of symptomatic PLE to corticosteroid therapy. However, due to the lack of a control group, the absence of untreated cases, and the fact that PLE was not systematically assessed in all patients (both symptomatic and asymptomatic), a definitive conclusion regarding the efficacy of corticosteroid treatment cannot be drawn.

The variability in clinical presentations and timing of symptom onset, spanning days to several months postoperatively, highlights the dynamic nature of PLE and its potential progression. This temporal variability demands prolonged and systematic follow-up, as early detection is paramount to effective intervention. The cases also emphasize the influence of patient-specific factors and systemic comorbidities in modulating the severity and trajectory of PLE. For instance, the presence of diabetes mellitus and chronic obstructive pulmonary disease in Case 5 likely compounded the patient's vulnerability to systemic and localized inflammatory responses, complicating the clinical course. Similarly, the pseudocyst formation and associated neurological deficits in Case 4 underscore the spectrum of PLE severity and the necessity for individualized management approaches. Sometimes the cystic formation could need stereotactic aspiration (13). Given the temporal variability of symptom onset, a symptom-driven, individualized follow-up protocol may be more appropriate than fixed imaging intervals.

These findings also raise important considerations regarding the predisposing factors for symptomatic PLE, including electrode positioning, surgical technique, and patient-specific immunological or vascular susceptibilities (6). The observed predominance of right-sided PLE may be incidental, but differences in venous anatomy, electrode trajectory, or handedness-related neuroanatomy could potentially play a role. Further investigation is warranted. The relatively high rate of severe PLE in our series may reflect specific patient susceptibilities or institutional differences in detection thresholds. All procedures were performed by the same experienced team, using consistent surgical techniques and the same electrode model. Further multicenter comparisons may help clarify whether technical or anatomical factors contribute to such presentations. Despite these challenges, the favorable outcomes achieved through corticosteroid therapy across all cases reaffirm the reversibility of PLE with timely intervention (14). Importantly, the resolution of symptoms and successful initiation of stimulation therapy post-edema resolution highlight the potential to achieve optimal therapeutic outcomes, even in the presence of initial complications (3).

While the pathophysiology of peri-lead edema (PLE) remains incompletely understood, recent literature suggests a multifactorial etiology involving procedural, anatomical, and patient-specific risk factors. One prospective study emphasized that PLE may be underreported due to the absence of systematic postoperative imaging, leading to missed subclinical cases (6). A comprehensive review and meta-analysis highlighted the lack of standardized diagnostic and follow-up protocols, underscoring the need for harmonized imaging strategies across centers (1). Procedural variables have also been implicated: an increased incidence of radiologic edema has been observed following asleep DBS procedures, suggesting that intraoperative factors such as altered venous drainage or cerebral perfusion may contribute to PLE development (15). Furthermore, reduced cortical and grey matter volumes on preoperative volumetric MRI have been associated with a higher risk of PLE, indicating that structural brain characteristics might predispose patients to this complication (3). Although our series did not systematically assess these variables, their integration into future risk stratification models may enhance preoperative planning and postoperative monitoring for high-risk individuals.

The implications of this case series extend beyond individual patient outcomes to inform broader DBS practice. Postoperative imaging, when guided by clinical symptoms, can facilitate early identification of symptomatic PLE, while targeted anti-inflammatory regimens can mitigate its impact (6). Moreover, further research is essential to elucidate the mechanistic underpinnings of PLE, optimize prevention strategies, and develop risk stratification tools. As DBS continues to expand as a therapeutic modality for neurological disorders, addressing complications such as symptomatic PLE will be critical to enhancing patient safety and ensuring the long-term success of this intervention (15).

## Conclusion

This study underscores the clinical significance of symptomatic peri-lead edema (PLE) as a rare but impactful complication in patients undergoing deep brain stimulation (DBS). The cases presented underscore the potential for severe manifestations of PLE requiring prompt identification and tailored intervention. The favorable outcomes observed following corticosteroid therapy suggest that anti-inflammatory treatment may be beneficial, though controlled studies are needed to establish causality.

These findings emphasize the need for vigilant postoperative monitoring, routine imaging, and individualized management to ensure optimal patient outcomes. The variability in presentations and the role of patient-specific factors, such as comorbidities and systemic health conditions, underline the complexity of this condition and the necessity for ongoing research to better understand its pathophysiology and risk factors. By integrating these insights into clinical practice, the safety and efficacy of DBS can be further enhanced, paving the way for improved care in the management of neurological disorders.

## Data Availability

The raw data supporting the conclusions of this article will be made available by the authors, without undue reservation.
